# Occurrence of symptoms and depressive mood among working-aged coronary heart disease patients

**DOI:** 10.1186/1477-7525-2-60

**Published:** 2004-11-08

**Authors:** Markku PT Sumanen, Sakari B Suominen, Markku J Koskenvuo, Lauri H Sillanmäki, Kari J Mattila

**Affiliations:** 1 Kangasala Health Centre, Finland; 2 Department for Social and Health Services, University of Turku, Finland; 3 Department of Public Health, University of Turku, Finland; 4 Medical School, University of Tampere and Department of General Practice, Hospital District of Pirkanmaa, Finland

## Abstract

**Background:**

The typical symptoms of coronary heart disease (CHD), chest pain and breathlessness, are well-known. They are considered quite dramatic, and can thus be fairly reliably mapped by a survey. However, people might have other clearly unpleasant symptoms impairing quality of life. The aim of this study is to evaluate the appearance of these complaints of working-aged people with self-reported CHD.

**Methods:**

The study consists of a postal questionnaire of randomly selected Finns in age groups 30–34, 40–44 and 50–54, a response rate of 39% (N = 15,477). The subjects were asked whether or not a doctor had told them that they had angina pectoris or had had myocardial infarction. Four randomly selected age and sex matched controls were chosen for every patient. The occurrence of self-reported dyspnoea, chest pain during anger or other kind of emotion, palpitation and perspiration without physical exercise, irregular heartbeats, flushing, trembling of hands and voice, jerking of muscles, depression and day-time sleepiness were examined. Odds ratios (OR) with 95% confidence intervals (CI), between occurrence of symptoms and CHD with and without heart infarction, were computed by multivariate logistic regression analysis.

**Results:**

The sample eventually comprised 319 CHD patients. Dyspnoea, chest pain during anger or other kind of emotion, palpitation, perspiration without physical exercise, irregular heartbeats daily or almost daily, trembling of hands and voice, and jerking of muscles occurred statistically significantly more frequently among CHD patients than among controls. The CHD patients also reported more depressive mood according to Beck's inventory scores and poorer sleep and more frequent day-time sleepiness than controls. In the multivariate logistic regression analysis chest pain during anger or other kind of emotion (ORs 4.12 and 3.61) and dyspnoea (ORs 2.33 and 3.81) were the symptoms most associated with CHD.

**Conclusions:**

Working-aged people with self-reported coronary heart disease evince a number of symptoms limiting the quality of their every day life. This aspect should be paid attention to when evaluating functional capacity of these patients.

## Background

In Finland, as in most industrialised countries mortality from cardiovascular diseases has shown decreasing trends since around 1970 [1,2]. The Mini-Finland survey from the years 1979–80 revealed that the angina pectoris symptom (i.e. reported chest pain under physical strain) may already appear in both sexes at the age of 30, though it was not until the age of 65 that it becomes more common among men compared to women [3].

The typical symptoms of coronary heart disease (CHD), chest pain and breathlessness, are well-known. They are considered quite dramatic, and thus can be fairly reliably mapped by a survey [4]. However, coronary heart disease patients also have other complaints in respect of their health, for example fatigue and sleep problems [5]. It has also been estimated that 17% to 27% of patients with coronary artery disease have major depression and a significantly larger percentage has subsyndromal symptoms of depression [6].

The diagnosis of CHD is usually based on medical examinations or register data and not on what people by themselves experience. However, people act and use health services according to what they experience to suffer from and what they experience to limit their ability to work. Since CHD patients have many other symptoms than the traditional and well-known, it is important to know what the spectrum of symptoms and complaints among working-aged people with self-reported coronary heart disease is in relation to functional capacity.

## Methods

### Design

The Health and Social Support study (HeSSup) is a prospective etiological follow-up study on the psychosocial health of the Finnish working-aged population. The HeSSup population consisted of a random sample of 39,563 individuals drawn from the Finnish Population Register in three age groups: 30–34, 40–44, and 50–54. The survey was carried out by postal questionnaire. Forms were returned by 15,477 individuals (approximately 5,000 in each age group), a response rate of 38.9% (37.6% in 30–34, 37.9% in 40–44, and 41.1% in 50–54).

The sample was subjected to a thorough analysis of non-response [7]. The analysis was made using the official statistics of the Finnish population for the corresponding age groups in 1998 to assess whether the study population adequately represented the Finnish population. Diagnosed epilepsy and diagnosed hypertension were selected to represent chronic diseases. The major reasons for refuse were the length of the questionnaire and above all suspicion of the purpose behind the request for written consent. Less educated, divorced, widowed, unemployed and those on disability pension were least willing to participate. Differences in physical conditions between the study participants and the whole population were, however, small. It was also noted that people suffering from hypertension returned the questionnaire somewhat less readily than others.

### Material and methods

The subjects were asked whether or not a doctor had told them that they had angina pectoris or had had myocardial infarction. The perceived state of health was determined according to Likert's five-step scale (good, quite good, fair, rather poor and poor). In order to avoid small frequencies in the analyses this was modified to a three-step scale (good, fair, poor).

The appearance of dyspnoea was categorised into four degrees of difficulty according to a widely used cardiovascular survey method [4]. Persons who suffered from dyspnoea when walking uphill or upstairs comprised the group of mild symptoms. Those out of breath when walking on level ground at normal speed with other people of the same age comprised the intermediate group, those who had to stop walking on level ground due to breathlessness comprised the group of difficult dyspnoea and those who became breathless even while standing still or while washing or dressing themselves, comprised the group of extremely difficult symptoms.

Participants were also asked whether or not they had experienced daily or weekly chest pain during anger or other kind of emotion, palpitation and perspiration without physical exercise, flushing, trembling of hands and voice, and jerking of muscles. Irregular heartbeats daily or weekly were asked, too. Depression was estimated by Beck's [8] depression scale ranging from 0 to 63. The normal score on this scale is below 10. In mild depression the scores are between 10 and 19 [9]. It was also asked how well and how many hours a day the participants had usually slept and how often they had felt day-time sleepiness, which when occurring daily or almost daily has been proved to be associated with depression, insomnia and breath interruptions during sleep [10].

### Analyses

In interpretation of results the coronary heart disease (CHD) patients were divided into two groups. The first comprised coronary patients not having had heart infarction (angina pectoris group) and the second patients having had heart infarction (infarction group). In order to have the best available comparison groups, four randomly selected age and sex matched controls for comparison were selected for every patient. Thus there were altogether 740 controls for the angina pectoris group and 536 controls for the infarction group. Stroke was ruled out in the control groups, but otherwise there were no differences between the CHD groups and their respective controls.

The associations, odds ratio (OR) with 95% confidence intervals (CI), between symptoms and coronary heart disease with and without heart infarction, were computed by multivariate logistic regression analysis. The analyses were made using the SAS System for Windows, release 8.2/2000.

## Results

The data comprised 319 patients: 185 coronary heart disease patients who had not experienced heart infarction (55.1% were men) and 134 patients who had (78.4% were men) (Table 1). Most of the CHD patients were in the oldest age group, and almost 90% of those who had had a heart infarction were in the age group 50–54. In all age groups the prevalence of self-reported CHD was higher among men than among women (Table 2).

**Table 1:** The coronary heart disease patients studied according to age and gender

	Angina pectoris group				Myocardial infarction group			
	
	Women	Men	Total		Women	Men	Total	
	
Age group	N	N	N	%	N	N	N	%
30–34	16	14	30	16	4	3	7	5
40–44	20	28	48	26	3	9	12	9
50–54	47	60	107	58	22	93	115	86
Total	83	102	185	100	29	105	134	100

**Table 2:** Prevalence of self-reported coronary heart disease according to age and gender

	Angina pectoris		Myocardial infarction	
	Women	Men	Women	Men
Age group	%	%	%	%
30–34	0.5	0.7	0.1	0.2
40–44	0.7	1.4	0.1	0.4
50–54	1.7	2.8	0.7	4.0

### Perceived state of health

State of health was perceived as good or quite good by 37.3% in the angina group and by 24.6% in the infarction group. The corresponding figures in the control groups were 76.0% and 67.4%, the differences being statistically significant (p < 0.001). State of health was perceived as poor or rather poor by 28.1% in the angina group and by 32.8% in the infarction group.

### Symptoms and complaints

At least mild breathlessness occurred in two thirds of the angina group and three fourths of the infarction group (Table 3). Difficult or extremely difficult breathlessness was reported by 20.2% in the angina group and by 27.8% in the infarction group. The corresponding figures in the control groups were 1.4% and 4.0%, the differences also being statistically significant (p < 0.001). Chest pain during anger or any kind of emotion, palpitation and perspiration without physical exercise, irregular heart beats, and jerking of muscles were all both daily and weekly statistically significantly more common among CHD patients than among controls. Almost daily CHD patients reported more daytime sleepiness and trembling of hands and voice than controls. CHD patients also slept more poorly than controls, and sleeping hours ≤ 6 in a day was more common among then than among controls. CHD patients scored higher on the depression scale than the controls, the average score being 10.2 (95% CI 9.0–11.4) in the angina group and 5.8 (95% CI 5.4–6.2) in the control group. In the infarction group the average score was 9.7 (95% CI 8.4–11.0). The corresponding figure in the control group was 5.9 (95% CI 5.4–6.5). In both coronary heart disease groups at least mild depression was twice as common as among controls. Flushing was the only complaint, which was not statistically significantly more common among CHD patients than among controls.

**Table 3:** Occurrence (%) of symptoms and complaints in coronary heart disease (CHD) patients and the control population

	Angina pectoris	Controls		Myocardial infarction	Controls	
	
	N = 177–185	N = 728–736		N = 129–134	N = 514–525	
	
	%	%	p	%	%	p
At least mild dyspnoea (Rose and Blackburn 1968)	66.5	33.2	<0.001	75.4	35.3	<0.001
Chest pain during anger or emotion						
Almost daily	12.2	1.0	<0.001	16.3	0.8	<0.001
Weekly	12.8	2.5	<0.001	16.3	3.3	<0.001
Palpitation without physical exercise						
Almost daily	14.1	3.4	<0.001	20.8	3.5	<0.001
Weekly	15.3	5.2	<0.001	16.9	5.4	<0.001
Perspiration without physical exercise						
Almost daily	22.7	9.7	<0.001	26.0	11.0	<0.001
Weekly	18.2	10.2	0.003	16.8	8.3	0.001
Irregular heart beats						
Almost daily	15.7	3.5	<0.001	24.0	3.7	<0.001
Weekly	12.4	6.1	0.004	14.0	4.4	<0.001
Depression (Beck ≥ 10)	41.6	20.4	<0.001	43.3	21.0	<0.001
Sleeping hours ≤ 6 in a day	16.8	10.3	0.015	19.4	10.1	0.003
Poor sleep usually	25.0	15.4	0.002	32.1	13.0	<0.001
Daytime sleepiness						
Almost daily	33.2	11.2	<0.001	32.1	14.9	<0.001
Flushing						
Almost daily	11.1	7.0	0.066	10.9	7.6	0.228
Weekly	12.8	7.1	0.014	9.3	8.0	0.624
Trembling of hands						
Almost daily	13.8	2.3	<0.001	9.2	3.3	0.004
Weekly	8.3	4.7	0.053	10.7	4.0	0.003
Trembling of voice						
Almost daily	3.3	1.1	0.030	5.3	1.2	0.002
Weekly	3.3	1.2	0.049	3.8	2.3	0.341
Jerking of muscles						
Almost daily	13.7	2.7	<0.001	12.3	4.4	0.001
Weekly	11.5	2.7	<0.001	9.2	3.8	0.011

### ORs of reported symptoms

In the multivariate logistic regression analysis chest pain during anger or other kind of emotion and dyspnoea were the symptoms most associated with CHD (Table 4). Irregular heart beats and perspiration without physical exercise were also strongly associated with heart infarction, but not with CHD without heart infarction. On the other hand, jerking of muscles was strongly associated with CHD without heart infarction, but not with heart infarction.

**Table 4:** Age- and sex-matched ORs with 95% CI in the multivariate logistic regression analysis for reported symptoms of coronary heart disease (CHD) without and with heart infarction. All of these symptoms were in the same model. Statistically significant associations are bolded.

	Angina pectoris	Myocardial infarction
	
	OR (95% CI)	OR (95% CI)
Dyspnoea	**3.81 (2.16–6.72)**	**2.33 (1.21–4.50)**
Chest pain during anger or emotion*	**3.61 (1.68–7.77)**	**4.12 (1.72–9.84)**
Palpitation without physical exercise*	1.19 (0.55–2.59)	1.39 (0.57–3.39)
Irregular heart beats*	1.46 (0.69–3.08)	**3.12 (1.28–7.60)**
Perspiration without physical exercise*	1.44 (0.87–2.38)	**2.19 (1.15–4.14)**
Flushing*	1.11 (0.62–1.97)	2.02 (0.89–4.59)
Trembling of hands*	1.36 (0.58–2.93)	1.01 (0.37–2.67)
Trembling of voice*	2.58 (0.85–7.87)	1.17 (0.29–4.69)
Jerking of muscles*	**2.44 (1.20–4.96)**	1.37 (0.55–3.41)
Depression (Beck ≥ 10)	1.57 (0.99–2.48)	1.01 (0.52–1.98)
Poor sleep	1.30 (0.74–2.27)	1.39 (0.70–2.77)
Daytime sleepiness	1.24 (0.76–2.01)	1.41 (0.78–2.56)
Sleeping hours ≤ 6 in a day	1.21 (0.64–2.26)	1.08 (0.50–2.33)

* almost daily or weekly

## Discussion

The principal finding in this study was that many working-aged coronary heart disease patients experience unpleasant symptoms such as dyspnoea, chest pain during anger or emotion, irregular heart beats, perspiration without physical exercise, and jerking of muscles. In addition, the frequency of most of the self-reported symptoms among the study population is higher also in respect of those symptoms, which would not be expected at least among those CHD patients whose disease is in good balance. Working-aged CHD patients may be regarded as a special group compared with the main part of CHD patients who are already by age entitled to a pension. It is likely that working-aged CHD patients have experienced the most widespread and intense exposure to risk factors, which thus has caused them this disease among the first ones within their age group. As working-aged they are wished, however, to return back to normal life and work as soon as possible. According to our study they still have a lot of symptoms concerning their every day life, which harm their recovery and rehabilitation. It is also noteworthy that many of the working aged CHD patients are still in working life. Although chest pain and dyspnoea do not prevent them to work at customer service, many of the symptoms such as trembling of hands interfere their normal jobs while appearing mostly in rest. Trembling of hands and voice are also very irritating symptoms, and they may be considered shaming. Thus they may interfere social life and reduce the quality of life.

The study material may be considered representative of the Finnish working-aged population, although the response rate was only 39%. Careful non-response analysis indicated that respondents and non-respondents were comparable in respect of the most important demographic variables [7]. It is possible that CHD patients respond to the questionnaire more actively than other people. On the other hand, there are certainly those among CHD patients who neglect their disease and are not willing to respond. However, we do not know for sure whether there is an over or under estimation of the associations, but we can presume that these two factors compensate each other. Moreover, it is unlikely that the principal association studied, i.e. the association between CHD and appearance of symptoms would be a substantially different one in non-participants. The findings reflect the respondents' own conception of their symptoms. The own conception of symptoms is important, since according to findings from a 3 years' follow-up of 4,000 men, self-reported coronary heart disease predicts very strongly a new coronary event [11]. In addition, the presence of anginal symptoms may be an important independent correlate of prognosis in patients with CHD [12].

Our method to determine the existence of CHD is based on the patients' report on whether a doctor had told them that they suffered from this particular disease. Thus, we cannot know for sure the accuracy of the information reported. Nowadays, CHD is rare among young people [1,2]. In our data there still is some. It is possible that there is a combination of several risk factors in the background.

There are validated instruments, such as the generic The Short Form 36 Health Survey [13] (SF-36), the Nottingham Health Profile [14], and the Seattle Angina Questionnaire [15], to investigate health-related quality of life. Our method to examine the subject was to ask about complaints and symptoms. Most of these questions have been successfully used in cardiovascular surveys [4] and in the Finnish Twin Cohort Study [16]. In addition, mood was estimated according the Beck's depression scale [8].

The occurrence of dyspnoea and chest pain even during anger or other kind of emotion may be considered a finding that was expected. In an American study on the care of coronary heart patients at the emergency department the most frequently reported symptom was chest pain (70% among men and 71% among women) and dyspnoea (30% men and 29% among women) [17]. The typical chest pain is also a symptom more predictive of an acute coronary attack in working-aged than in older patients [18].

It was suggested as far back as 1987 that palpitations are not an independent risk factor for increased cardiac morbidity or mortality [19]. However, according to a British study those experiencing palpitations at work and while asleep were more likely to have a cardiac cause for their palpitations [20]. Our finding was that working-aged CHD patients report palpitations more often than the control population.

The high occurrence of trembling of hands and voice, and jerking of muscles may be considered an unexpected finding. Most CHD patients use beta-blocking medicines, which in addition to protecting the heart muscle also reduce the adrenergic stimulation and thus relief the symptom of trembling.

Concerning depression our findings support those of previous studies. One out of four of symptomatic coronary heart disease patients have namely been found to have a probable depressive disorder, but none of them had previously been identified as suffering from depression or been treated for this reason [21]. In primary care it is of vital importance to notice symptoms of this illness, since continuing depression has been found to be associated with increased risk of mortality among CHD patients following hospital discharge [22]. It has also been verified that depression is common after coronary heart disease events such as bypass grafting, coronary angioplasty, myocardial infarction and myocardial ischaemia [23]. In our study about 40% of CHD patients had at least minor depression compared with 20% among controls. The high depression rates are probably due to our method to diagnose depression at ≥ 10 points in Beck's inventory scale. Thus we do not think there are any selection bias, since the controls were randomly selected. Furthermore, it is not probable that depressed people respond to our questionnaire more readily than people not suffering from low mood.

It was also of noteworthy that daytime sleepiness was connected with coronary heart disease. A cross-sectional study of 5,419 Finnish adult men found a higher prevalence of diagnosed myocardial infarction among those who slept more than nine hours, whilst those sleeping less than six hours per night had more symptomatic coronary disease [24]. In a Swedish study concerning working-aged women poor sleep was associated with an increase in spasmodic chest pain and irregular heart beat [25], whereas in men an association between difficulties falling asleep and CHD mortality has been found [26].

From previous research we know that despite having survived a life-threatening clinical event, CHD patients appear to have continued adverse behaviours such as smoking, being obese and having frequent hangovers more than the control population [27]. The follow-up of our cohort will show in what extent the symptoms we found are indicators of increased risk of CHD among working-aged people and to what extent the symptoms are result of CHD and its care. In both cases particular attention should be paid to these aspects in primary care.

## Conclusions

According to the present findings many working-aged people with self-reported coronary heart disease perceive their state of health as poor or rather poor. They suffer from a wide range of symptoms limiting their every day life. It is noteworthy that many of these symptoms are not only irritating, but constitute a threat to health. The health related quality of life is poor among working-aged coronary heart disease patients.

## Competing interests

The author(s) declare that they have no competing interests.

## Authors' contributions

MPTS drafted the manuscript; SBS participated in drafting of the manuscript; MJK participated in the design of the study and the statistical analyses; LHS participated in the statistical analyses; KJM conceived of the study, and participated in its design and co-ordination. All authors have read and approved the final manuscript.
